# Expression of CD150 in Tumors of the Central Nervous System: Identification of a Novel Isoform

**DOI:** 10.1371/journal.pone.0118302

**Published:** 2015-02-24

**Authors:** Olga Romanets-Korbut, Alexander M. Najakshin, Mariya Yurchenko, Tatyana A. Malysheva, Larysa Kovalevska, Larysa M. Shlapatska, Yuriy A. Zozulya, Alexander V. Taranin, Branka Horvat, Svetlana P. Sidorenko

**Affiliations:** 1 Laboratory of signal transduction pathways, R.E. Kavetsky Institute of Experimental Pathology, Oncology and Radiobiology of NAS of Ukraine, Kyiv, Ukraine; 2 CIRI, International Center for Infectiology Research, IbIV team, Université de Lyon, Lyon, France; 3 Inserm, U1111, Lyon, France; 4 CNRS, UMR5308, Lyon, France; 5 Université Lyon 1, Lyon, France; 6 Ecole Normale Supérieure de Lyon, Lyon, France; 7 Laboratory of immunogenetics, Institute of Molecular and Cellular Biology of SB RAS, Novosibirsk, Russia; 8 Neuropathomorphology Department, A.P. Romodanov Institute of Neurosurgery NAMS of Ukraine, Kyiv, Ukraine; 9 Novosibirsk State University, Novosibirsk, Russia; Swedish Medical Center, UNITED STATES

## Abstract

CD150 (IPO3/SLAM) belongs to the SLAM family of receptors and serves as a major entry receptor for measles virus. CD150 is expressed on normal and malignant cells of the immune system. However, little is known about its expression outside the hematopoietic system, especially tumors of the central nervous system (CNS). Although CD150 was not found in different regions of normal brain tissues, our immunohistochemical study revealed its expression in 77.6% of human CNS tumors, including glioblastoma, anaplastic astrocytoma, diffuse astrocytoma, ependymoma, and others. CD150 was detected in the cytoplasm, but not on the cell surface of glioma cell lines, and it was colocalized with the endoplasmic reticulum and Golgi complex markers. In addition to the full length mRNA of the mCD150 splice isoform, in glioma cells we found a highly expressed novel CD150 transcript (nCD150), containing an 83 bp insert. The insert is derived from a previously unrecognized exon designated Cyt-new, which is located 510 bp downstream of the transmembrane region exon, and is a specific feature of primate *SLAMF1*. Both mCD150 and nCD150 cDNA variants did not contain any mutations and had the leader sequence. The nCD150 transcript was also detected in normal and malignant B lymphocytes, primary T cells, dendritic cells and macrophages; however, in glioma cells nCD150 was found to be the predominant CD150 isoform. Similarly to mCD150, cell surface expression of nCD150 allows wild type measles virus entry to the cell. Our data indicate that CD150 expression in CNS tumors can be considered a new diagnostic marker and potential target for novel therapeutic approaches.

## Introduction

CD150 (IPO3/SLAM, for Signaling Lymphocytic Activation Molecule) is a membrane protein that belongs to the SLAM family within the immunoglobulin superfamily of surface receptors. In humans it is encoded by the *SLAMF1* gene [1–3]. CD150 is mainly expressed within hematopoietic cell lineage: on thymocytes, activated T and B lymphocytes, dendritic cells, macrophages and activated monocytes [3–8]. CD150 was also found on malignant cells of lymphoid origin [9]. However, little is known about CD150 expression outside of the hematopoietic system, particularly in tumors. In addition to the transmembrane form of CD150 (mCD150), cells of hematopoietic lineage express mRNA encoding the secreted form of CD150 (sCD150), which lacks the entire transmembrane region of 30 amino acids [4,10,11]. They also express mRNAs of the cytoplasmic form (cCD150) lacking the leader sequence, and a variant membrane CD150 (vmCD150 or tCD150) with a truncated cytoplasmic tail [12]. Nevertheless, expression of the vmCD150 isoform was not confirmed at the mRNA level [11].

CD150 receptor is a self-ligand and functions as a co-receptor molecule that regulates signaling via antigen receptors [13]. It is also associated with several components of the bacterial killing machinery, which defines it as a novel bacterial sensor [14,15]. Moreover, CD150 was found to be the major receptor for several *Morbilliviruses*, including measles virus (MV), canine distemper virus (CDV) and rinderpest virus [16,17]. MV infection could be associated with numerous complications, of which especially severe are those that involve the central nervous system (CNS), as MV is implicated in the pathogenesis of several types of encephalitis [18]. However, currently identified cellular receptors for wild type MV make the virus entry possible only in lymphoid cells (through CD150) or epithelial cells (through nectin-4) [16,19,20]. The third MV receptor, CD46, is expressed on all human nucleated cells and is able to mediate the entry of laboratory adapted and vaccine strains of the virus [21,22]. Thus, it is still unknown how MV enters the cells of the nervous system.

MV attracts much interest as a potential oncolytic virus [23,24]. It showed a potent antitumor activity against different tumor cell lines and was used in oncolytic therapy of such highly malignant CNS tumors as gliomas [25,26].

Here we show that CD150 is expressed in CNS tumors and glioma cell lines. This antigen is not expressed on the surface of the tested glioma cells, but was found in the cytoplasm where it was colocalized with endoplasmic reticulum and Golgi complex. In addition to the conventional splice isoform, we identified a novel CD150 isoform (nCD150) with an 83 bp insert, which is derived from a previously unrecognized exon downstream of the exon for the transmembrane domain, and is a specific feature of the primate CD150 gene. The shift of the “conventional” reading frame results in the production of nCD150 with a cytoplasmic tail that lacks any known signaling motifs. The nCD150 transcript is expressed in cells of different origin, but in glioma cells it is the predominant CD150 isoform. Since CD150 is not revealed in normal brain tissues but is expressed in 77.6% of CNS tumors, CD150 could be a novel diagnostic marker for CNS tumors and a potential target for the therapy of gliomas.

## Materials and Methods

### Ethics Statement

Written informed consent from each patient was obtained, and all experimental procedures were performed in accordance with the Declaration of Helsinki. The usage of biopsy specimens was approved by the Institutional Review Board and Research Ethics Committees of R.E. Kavetsky Institute of Experimental Pathology, Oncology and Radiobiology, NAS of Ukraine and A.P. Romodanov Institute of Neurosurgery of AMS of Ukraine.

### Cell lines

U87 cell line was purchased from ATCC. Primary glioblastoma cell lines from adult patients in the advanced stage of disease NCH89 and NCH92 [27], glioma cell lines A172, U343 and TE671 (Tumorbank, DKFZ, Heidelberg, Germany) were kindly provided by Dr. Karsten Geletneky (Department of Neurosurgery, University Hospital Heidelberg, Germany) and Prof. Joerg R. Schlehofer (Department of Applied Tumor Virology, German Cancer Research Center, Heidelberg, Germany). The glioma cell lines A172, U87, U343, TE671, NCH89, NCH92, human embryonic kidney fibroblasts HEK293T (ATCC) and Vero-SLAM cell line were maintained in DMEM medium containing 10% FCS, 2 mM L-glutamine, and antibiotics. Burkitt’s lymphoma cell line BJAB (from DSMZ), B lymphoblastoid cell lines (LCL) MP-1 and T5–1 were kindly provided by Prof. Edward Clark (University of Washington, Seattle, WA, USA). Hodgkin’s lymphoma cell line L1236 cell line from DSMZ was kindly provided by Prof. Eva Klein (Karolinska Institute, Stockholm, Sweden). Cell lines BJAB, L1236, MP-1 and T5–1, human acute monocytic leukemia cell line THP-1 (from ATCC) and pre-B acute lymphocytic leukemia cell line REH (from ATCC) were maintained in RPMI 1640 medium containing 10% FCS, 2 mM L-glutamine, and antibiotics.

### Human tissue specimens and primary human cells

Immunohistochemical analyses were performed on surgical specimens sections of human primary CNS tumors (108 cases) and human brain tissues from patients with non-tumor pathology that were obtained from the A.P. Romodanov Institute of Neurosurgery of AMS of Ukraine. CNS tumors were classified according to the World Health Organization (WHO) classification. Verification of the diagnosis was performed based on a combination of morphologic, topographic and clinical characteristics. Paraffin-embedded sections were stained with mouse anti-CD150 mAb IPO-3 (IgG1, clone IPO-3, IEPOR NASU, Kiev, Ukraine), rabbit anti-glial fibrillary acidic protein (GFAP) (DakoCytomation, DK) and anti-nestin (Sigma, USA) antibodies followed by the EnVision system (DakoCytomation, DK), developed with diaminobenzidine tetrahydrochloride (DAB) substrate (DakoCytomation, DK), and examined using Leica DC 150 microscope complex (Germany).

Tonsils were obtained from patients undergoing tonsillectomy. Tonsillar B cells were isolated by density fractionation on discontinuous Lymphoprep (Axis-Shield PoC AS, Norway) and Percoll (Sigma, USA) gradients as described [7]. Human peripheral blood was obtained from healthy donors from the Blood Transfusion Centre (Lyon, France). PBMC were isolated by density Ficoll/Hypaque gradient centrifugation, and then centrifuged through a 50% Percoll gradient (Pharmacia Fine Chemicals, Uppsala, Sweden) for 20 min at 400 g. Peripheral blood lymphocytes (PBLs) were recovered from the high density fraction and monocytes from the light density fraction at the interface. CD3^+^ and CD38^+^ lymphocytes were isolated, using microbeads (Miltenyi Biotech, Germany) and magnetic cell separation with MACS Separator. DCs were generated *in vitro* from the adherent fraction of purified monocytes, treated for 6 days at 5 x 10^5^ monocytes/ml with IL-4 (250U/ml, Peprotech, USA) and GM-CSF (500U/ml, Peprotech, USA). Macrophages were generated *in vitro* from the adherent fraction of purified monocytes, adjusted to the density of at 5 x 10^5^ monocytes/ml, and treated with M-CSF (250 U/ml, Peprotech, USA) for 6 days. Both CD1d^+^ DCs and T cells were further cultured in RPMI 1640 medium containing 10% fetal calf serum, 2 mM L-glutamine, 10mM HEPES and antibiotics.

### Splice isoforms cloning from U87 cells

mRNA was isolated from U87 cells using ToTally RNA kit (Ambion, USA). First strand cDNA was synthesized using RevertAid First Strand cDNA Synthesis Kit (Fermentas, USA) according to manufacturer’s instructions. cDNA was amplified using Fusion polymerase (Finnzymes, USA) and the following primers: 5’-catctcgagCCTTCTCCTCATTGGCTGATGG-3’ (329–350, CD150 mRNA sequence GI:176865712) as forward primer and 5’-cacgcggccGCAGCATGTCTGCCAGAGGAA-3’ (1436–1456) as reverse primer. The PCR fragments of CD150 splice isoforms were eluted from the gel with MiniElute Gel Extraction Kit (Qiagen, USA), digested by XhoI and NotI, and ligated into pCI-neo vector (Promega,USA). Transformation was performed using *E*.*coli* XL-Blue MRF’ electrocompetent cells and clones with inserts were selected and sequenced as described elsewhere.

### Reverse-Transcriptase PCR

Total RNA was isolated from cells using TRIzol reagent (Sigma-Aldrich, St. Louis, MO, USA) according to manufacturer’s instructions. 5 x 10^6^ cells of cell lines or primary cells were homogenized in 1 ml of TRIzol reagent, and processed according to the manufacturer’s instructions. Reverse transcriptase reactions were performed with RevertAid First Strand cDNA Synthesis Kit (Fermentas, USA). Obtained cDNAs were amplified by PCR using Taq DNA polymerase (Invitrogen, USA). Specific primers were used to detect distinct CD150 domains: for the extracellular CD150 domain ExtraCD150, 5’-ATGGATCCCAAGGGGC-3’ (347–362) as sense, and 5’-CCCAGTATCAAGGTGCAGGT-3’ (815–834) as antisense primers; for the transmembrane domain, TM CD150, 5’-ACAGACCCCTCAGAAACAAAACCAT-3’ (1034–1058) as sense, and 5’-CGTGCAGCATGTCTGCCAGAGGAAACTTG-3’(1438–1459) as antisense; for the cytoplasmic tail Cyt-mCD150, 5’-TTGAGAAGAAGAGGTAAAACGAAC-3’ (1124–1147) as sense and 5’-CTGGAAGTGTCACACTAGCATAG-3’ (1324–1346) as antisense; for the novel CD150 isoform nCD150, 5’-TGCTGACAATATCTACATCTG-3’ (952–972) as sense and 5’-CAGTATTGGTTGGTAGTAGTC-3’ (in Cyt-new exon) as antisense; for GAPDH, used for the evaluation of cDNA quality and quantity, 5’-TCATTATGCCGAGGATTTGGA-3’ as sense and 5’-CAGAGGGCCACAATGTGATG-3’ as antisense. PCR products were resolved in agarose gels and visualized after staining with ethidium bromide. For sequencing, PCR products were isolated from gel using the Quigen gel isolation kit (Quigen, USA). Alignment of sequenced PCR products with CD150 cDNA (gi:176865712) was performed using the nucleotide-nucleotide alignment option (blastn) in the BLAST internet program (http://blast.ncbi.nlm.nih.gov/Blast.cgi).

### Real-Time PCR

Total RNA was isolated from cells and tumor samples using TRI reagent (Sigma-Aldrich, St. Louis, MO, USA). Approximately 2*μ*g of total RNA was used for cDNA synthesis, using RevertAid Reverse Transcriptase, RiboLock RNase Inhibitor (Thermo Scientific, Lithuania), and Oligo(dT)23 anchored primer (Sigma-Aldrich, St. Louis, MO, USA) according to manufacturer’s protocol. cDNA obtained was used for real-time PCR. Real-time PCR was performed using Maxima SYBR Green/ROX qPCR Master Mix (Thermo Scientific, Lithuania) on 7500 machine (Applied Biosystems, Foster City, CA, USA). The following primers for real time PCR were used: CD150 Extr (isoforms containing extracellular part of CD150 receptor): For 5’-AGGCCCTCCACGTTATCTA-3’, Rev 5’- GCAAAAGCGCTGAACTGA-3’; Cyt-n (isoforms containing alternative cytoplasmic tail, nCD150): For 5’- TGAGAAGAAGAGCCACCTTGA-3’, Rev 5’- GGTTCGTTTTACCATGGGAAG-3’; Cyt-m (isoforms containing conventional cytoplasmic tail): 5’- TGAGAAGAAGAGGTAAAACGAACC-3’, Rev 5’- ATATGGTGGTGCAAGGGTCC-3’. PCR products were sequenced to control the specificity of reactions. As an internal control for standardization, we have assayed the expression of a gene encoding TBP—TATA-binding protein (*Tbp*, NM_003194 and NM_001172085.1). The following primers were used For 5’- GAGCCAAGAGTGAAGAACAGTC-3’, Rev 5’- GCTCCCCACCATATTCTGAATCT-3’. The PCR cycling conditions were the following: 10 min at 95°C, 40 cycles of 10s at 95°C and 1 min at 62°C. Applied Biosystems 7500 system software was used for analysis. Ct values were determined for the internal control (TBP) and the test genes at the same threshold level in the exponential phase of the PCR curves. Relative quantification (comparative Ct (ddCt) method) was used to compare the expression level of the test genes with the internal control. Expression level of mRNA coding for each CD150 isoform was expressed relative to respective isoform in CD38^+^ B cells, the value for which was set at 1. Dissociation curve analysis was performed after every run to check the specificity of the reaction. For each gene 3–5 reactions (in triplicates) were run and a SEM was calculated.

### Transfection of HEK293T and U87 cells

HEK293T cells were transfected with pRRLsin-CMV-IRES-mCD150 (kindly provided by Dr. Denis Gerlier, CIRI, Lyon, France) and pCI-neo-nCD150 plasmids using JetPRIME reagent (Polyplus transfection) following the manufacturer’s protocol. U87 cells were transduced using retroviruses containing retro vectors pBABE-mCD150 and pBABE-nCD150 based on pBABE-puro (Addgene, USA). The plasmids pHZT and pCPG were utilized for packaging in HEK293T cells for the production of retroviral particles.

### Flow cytometry

To study the expression of CD150, glioma cell lines were stained with mouse mAb followed with PE-conjugated goat anti-mouse IgG (BD Biosciences). Cells were acquired on FACSCalibur 3C cytometer (Becton Dickinson, USA) analyzed using CellQuestPro software followed by FlowJo data analysis software (Tree Star Inc., USA).

### Western blot analysis

Western blot analysis was performed as described [9] using anti-CD150 rabbit monoclonal antibodies (10837-R008, Sino Biologicals Inc., China) followed by goat anti-rabbit HRP-conjugated antibodies (sc-2054, Santa Cruz Biotechnology, USA).

### Cell staining

For immunostaining, adherent glioma cell lines and CHO-SLAM were grown on cover slips, while B cell line MP-1 cells were attached to the microscope slides using Cytospin centrifuge. All cell lines were fixed/permeabilized with methanol:acetone (1:1) for 1 h at -20°C followed by the overnight rehydration with PBS at +4°C. The incubation with primary antibodies lasted for 1 h, followed by three washes in PBS, secondary antibody incubation for 30 min, three washes in PBS and one brief wash in water. DAPI was added to the last secondary antibody for DNA staining.

For confocal microscopy the glioma cell line U87 and B cell line MP-1 were stained with rabbit anti-Kinectin (Santa Cruz Biotechnology, USA), or anti-GRP78, or anti-furin antibodies (Tebu-bio, France), followed by donkey anti-rabbit IgG conjugated with Alexa 647 (Molecular Probes, Invitrogen, USA), and anti-CD150 mouse monoclonal antibodies, followed by rabbit anti-mouse IgG antibodies labeled with Alexa 546 (Molecular Probes, Invitrogen, USA). DAPI (4’,6-diamidino-2-phenylindole) was added to the last secondary antibody for DNA staining. Finally, the microscope slides were analyzed using Zeiss LSM-710 fluorescence microscope equipped with Zeiss Zen software for immunofluorescence analysis and Leica SP5 for the confocal analysis. Colocalization was characterized by Manders overlap coefficient. Mander’s coefficient is a product of channel intensities that return a significant value only when both channel values belong to the same pixel. It indicates channel signal overlap and represents the true degree of colocalisation. Mander’s coefficient ranges from 0 to 1, where 0 is defined as no colocalization and 1 as perfect colocalization, and was calculated with the ImageJ (MacBiophotonics, USA) software. Results are presented as means ± SD.

### Cell infection with measles virus

The wild-type (wt) MV strain, G954 (genotype B3.2), was isolated in Gambia in 1993. The wt MV strain was propagated on Vero-SLAM cells. MV vaccine strain Edmonston was obtained from ATCC (VR-24). Viruses were titrated by assaying PFU on Vero-SLAM cell monolayers. For this, different dilutions were cultured on Vero-SLAM cell monolayers for 4 days of culture, fixed, and then stained with methylene blue. The number of lysed-cell dots corresponded to the number of plaque forming units and number of primary infectious viral particles applied. Glioma cell lines and Vero-SLAM cells were infected with either G954 or Edmonston MV strains at MOI of 1.

### Statistical analysis

The statistical analysis for the comparison between two groups was performed using unpaired two-tailed Student’s t test. Results are shown as mean ± SD or mean ± SEM.

## Results

### CD150 is expressed in tumors of the central nervous system

**Fig 1:**
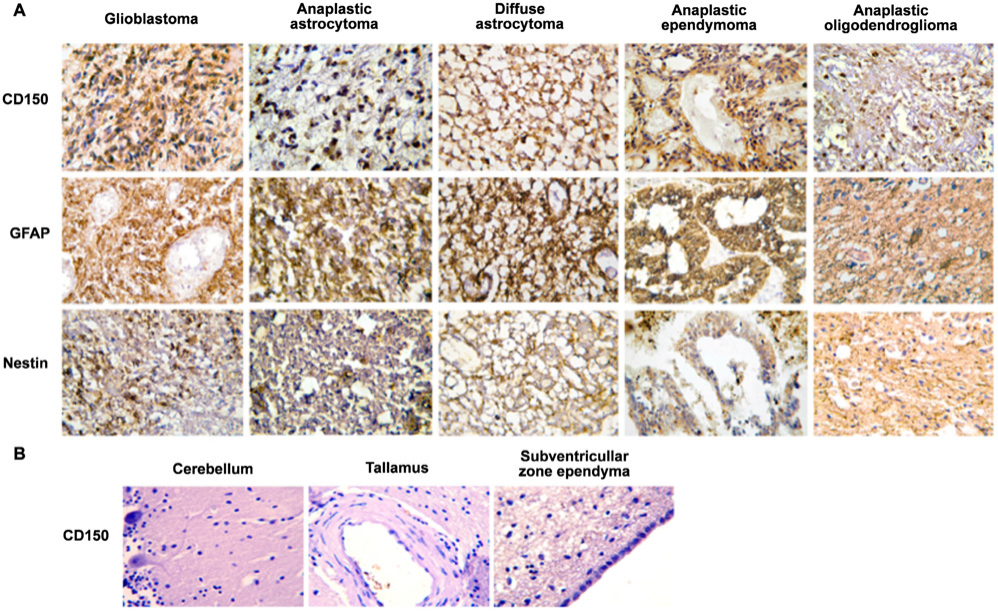
Expression of CD150 in human central nervous system (CNS) tissues. (A) Immunohistochemical analysis of CD150 expression in human primary CNS tumors. Tumor paraffin sections of glioblastoma, anaplastic astrocytoma, diffuse astrocytoma, anaplastic ependymoma and anaplastic oligodendroglioma were stained with antibodies to CD150 (upper panel), glial fibrillary acidic protein, GFAP, astrocyte marker (middle panel) and nestin, neural stem/progenitor cell marker (lower panel). CD150 expression was detected in all presented histological variants of CNS tumors (upper panel). (B) Expression of CD150 in different regions of human normal brain tissues: cerebellum, thalamus, subventricular zone ependyma. The expression of CD150 was not found in human normal brain tissues. DAB staining shows specific reactions in brown. Cell nuclei are lightly counterstained with haematoxylin. Microscopic magnification of 400× was used for all images.

To find out whether CD150 is expressed in brain tissues and tumors of the central nervous system we performed immunohistochemical studies of primary human CNS tumors (108 cases) and brain tissues from patients with non-tumor pathology. Our studies revealed CD150 expression in tumor cells (Fig. 1A). The level of expression and the number of positive cases varied for tumors of different histogenesis and grade (Table 1). Immunostaining of all samples with the antibodies to glial fibrillary acidic protein (GFAP) and nestin (neural stem/progenitor cell marker) (Fig. 1A) and the macrophage marker CD68 (data not shown) validated CD150 expression in malignant cells.

**Table 1:** CD150 expression in CNS tumors.

Tumor histological type	Number of cases	CD150-positive cases, %	Tumor* grade
Glioblastoma	18	88,9	IV
Anaplastic astrocytoma	16	81,3	III
Diffuse astrocytoma	12	66,7	II
Anaplastic ependymoma	6	75	III
Ependymoma	4	66,7	II
Anaplastic oligodendroglioma	8	100	III

* Tumor grade according to WHO classification

The highest level of CD150 expression was found in glioblastoma, which also had the highest incidence of CD150 positive cases (88.9% of cases) (Fig. 1A, Table 1). The product of immunohistochemical reaction was detected in the cytoplasm of 70–90% of tumor cells with staining of cell processes. Two CD150 negative glioblastoma cases were characterized by high levels of nestin and GFAP expression. Tumor cells of anaplastic astrocytoma also often expressed CD150 (81.3% of cases), however, they demonstrated low to moderate immunoreaction. In one case, CD150 expression was found only in giant tumor cells. Anaplastic astrocytoma cells expressed high levels of GFAP, while nestin expression was detected only in scattered cells (Fig. 1A). CD150 expression was found in the majority of tumor cells in 66.7% cases of diffuse astrocytoma. Positive cytoplasmic reaction was observed both in the perinuclear zone and in cell processes (Fig. 1A). The fibrillary matrix, which consists of a network of neoplastic cell processes, formed a diffuse CD150 positive background (Fig. 1A). GFAP was consistently expressed, and one third of cells showed immunostaining for nestin (Fig. 1A). The frequency of CD150 positive cases for ependymoma and anaplastic ependymoma was similar (66.7% and 75.0% respectively). Moderate to high immunostaining was observed in cytoplasm of tumor cells. These tumors were characterized by high level of GFAP as well as nestin expression (Fig. 1A). It should be noted that anaplastic oligodendroglioma (4 cases) demonstrated moderate to high levels of immunostaining for CD150 with a reaction not only in the cytoplasm, but also in the nucleus (Fig. 1A).

**Fig 2:**
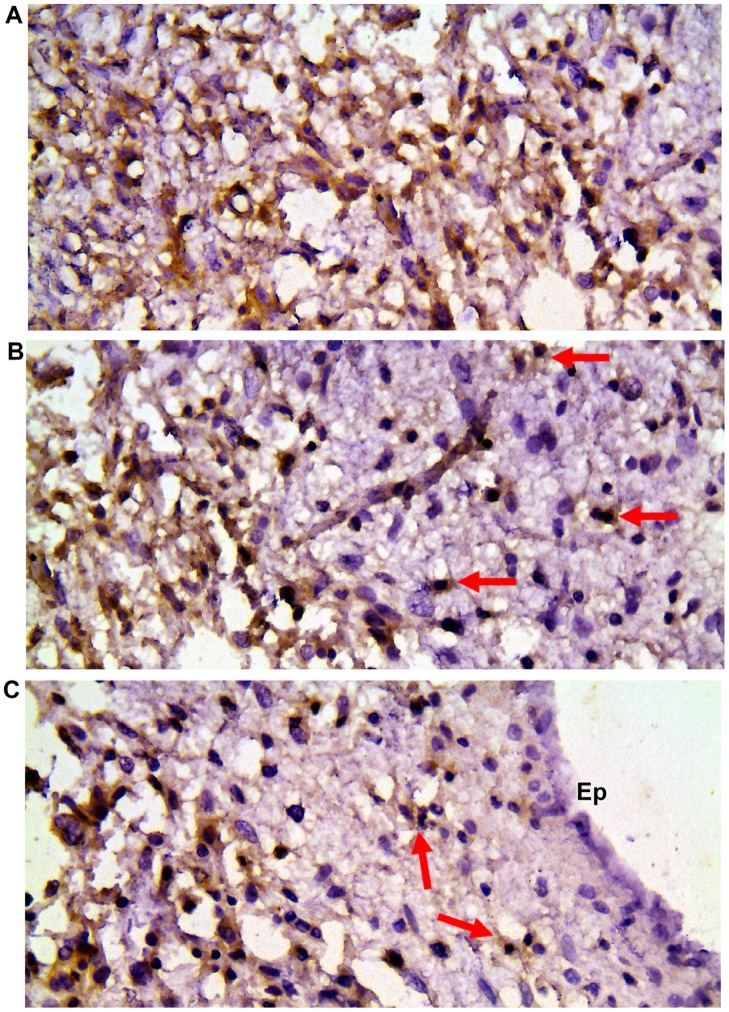
CD150 expression in serial sections of tumor and reactive brain tissues. (A) Diffuse astrocytoma with extensive microcyst formation. (B) Zone of infiltration with tumor cells of perifocal brain tissue. Scattered tumor cells are shown with arrows. Activated microglia and reactive astrocytes are CD150 negative. (C) Infiltration of subventricular zone with tumor cells (arrows), negative reaction in ependymal cells (Ep). DAB staining shows specific reaction in brown. Cell nuclei are counterstained with haematoxylin. Microscopic magnification 400×.

Expression of CD150 was also found in anaplastic oligoastrocytoma, anaplastic medulloblastoma, CNS primitive neuroectodermal tumour, subependymal giant cell astrocytoma (Tuberous sclerosis), CNS neuroblastoma, central neurocytoma. At the same time oligodendroglioma, medulloblastoma and dysembryoplastic neuroepithelial tumour were CD150-negative. To answer the question whether CD150 expression is a characteristic feature of CNS tumors, we also studied sections of seven different regions of brain tissues from patients with non-tumor pathology. The expression of CD150 in different human brain regions was not observed (Fig. 1B). Moreover, it is possible to track infiltration of zone of invasion with CD150^+^ tumor cells. It should be emphasized that activated reactive astrocytes in zone of tumor invasion do not express CD150 (Fig. 2).

Taken together, we found tumor cell-specific CD150 expression in 77.6% cases of CNS tumors. CD150 was detected mainly in the cytoplasm of tumor cells. Nevertheless, an immunohistochemical approach cannot determine if this antigen is expressed on the cell surface and can thus be used for measles virus entry.

### CD150 is expressed in the cytoplasm but not on the surface of human glioma cell lines

**Fig 3:**
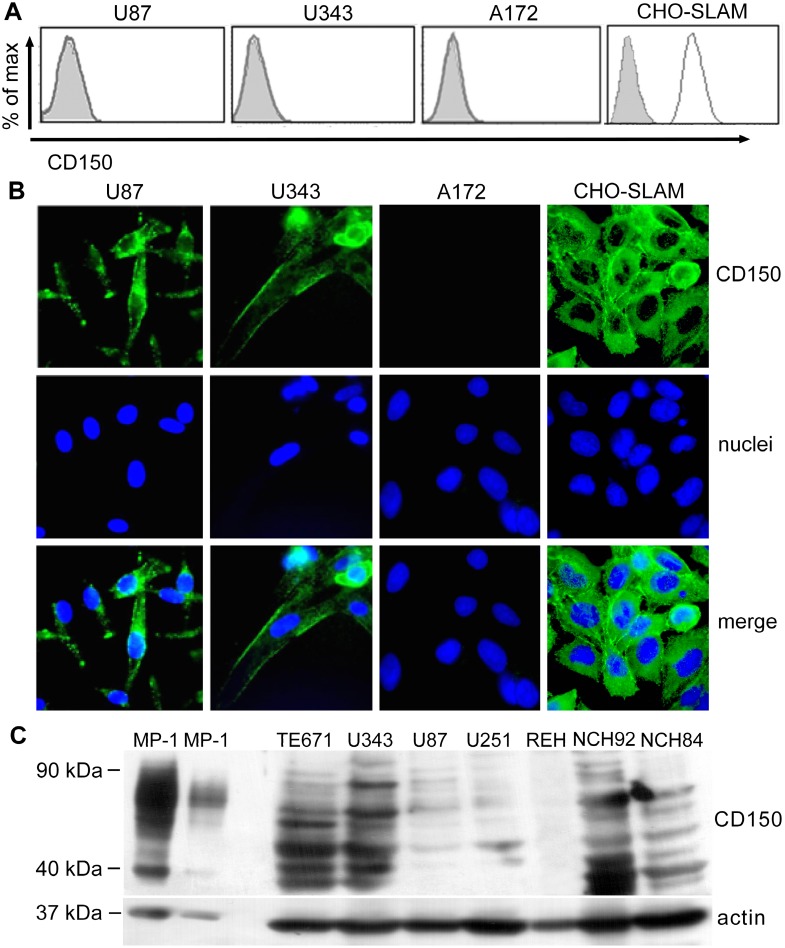
Expression of CD150 in human glioma cell lines. (A) Flow cytometry study of CD150 surface expression on U87, U343 and A172 cells. One of four experiments. (B) Immunofluorescent analysis of CD150 expression in the cytoplasm of U87, U343 and A172 glioma cell lines. The cells were fixed, stained with anti-CD150 antibodies (IPO-3) followed by secondary antibody labelled with Alexa 488 (upper panel). Nuclei were visualized by staining with DAPI (middle panel). Results are representative of more than five experiments. Magnification: 630×. (C) Western blot analysis of CD150 expression in glioma cell lines using rabbit monoclonal anti-CD150 antibodies (Sino Biologicals Inc., China). Pre-B cell line REH and B-LCL MP-1 (in two different dilutions) were used as negative and positive controls respectively. One of five experiments is presented. The flow cytometry analysis of live cells does not show any CD150 expression in all used glioma cell lines, however fluorescent studies of fixed permeabilized cells and western blot detected CD150 expression in U87, U343, U251, TE671, NCH84, and NCH92 glioma cell lines.

To clarify the localization of CD150 in glial tumor cells, we performed additional immunostaining of CD150 in glioma cell lines. Several glial cell lines (A172, U87, U343, TE671) and primary glioblastoma cell lines (NCH89 and NCH92) were stained with anti-CD150 antibodies and visualized using flow cytometry and fluorescent microscopy. We did not detect CD150 on the surface of these cells by flow cytometry (Fig. 3A). However, the immunofluorescent analysis of methanol-acetone fixed cells revealed CD150 expression in the cytoplasm of U87, U343, NCH89, NCH92, TE671, but not A172 cells (Fig. 3B). Staining of CHO SLAM cell line was used as the positive control. Western blot analysis confirmed the expression of CD150 protein in glioma cell lines, but the level of expression was much lower than in B-lymphoblastoid cell line (LCL) (Fig. 3C).

**Fig 4:**
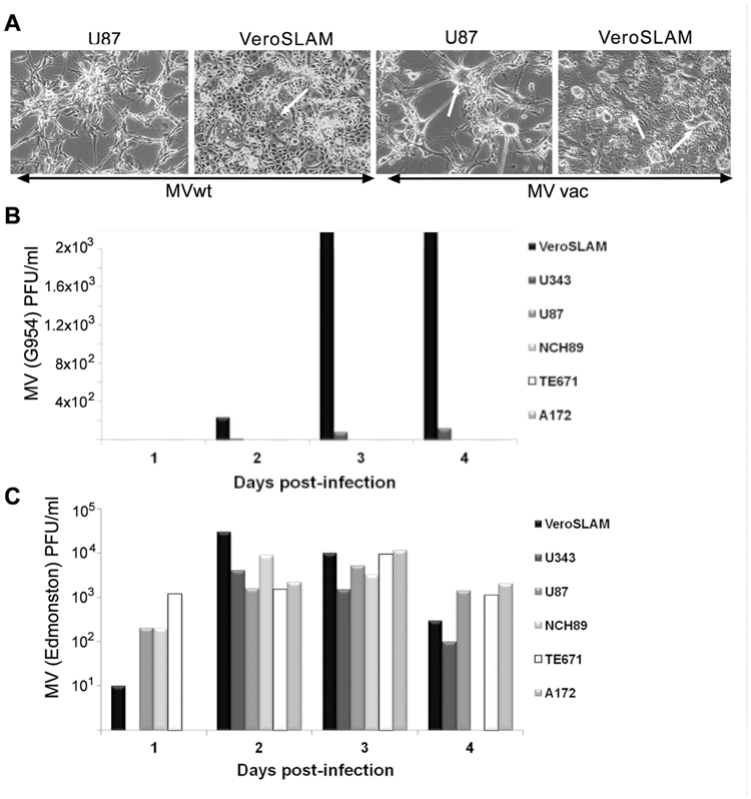
Measles virus (MV) does not infect human glioma cell lines. (A) Measles virus cytopathic effect in glioma and control cell lines. U87 glioma cell line and Vero-SLAM cells (positive control) were infected with wild type MV strain G954 and laboratory MV strain Edmonston, at MOI of 1. Images show syncytia formation (white arrows) at 24 h (for Vero-SLAM cells) or 96 h (for U87 cells) post-infection. Microscopic magnification of 400× was used for all images. One of five experiments. (B-C) Wild type (B) and laboratory strain (C) MV production in glioma and control cell lines, determined daily by plaque assay on Vero-SLAM cells, 24 h (1), 48 h (2), 72 h (3) and 96 h (4) post-infection. Vero-SLAM and Vero cells were used as positive and negative control respectively. One of three representative experiments. All studied glioma cell lines were not sensitive to the infection with wild type measles virus (MV), which uses CD150 receptor for its entry. At the same time all cell lines were infected and produced high titers of laboratory adapted Edmonston MV strain that also uses ubiquitously expressed CD46 as entry receptor.

Since CD150 cell surface expression could be below the level of detection by immunofluorescence, we tested glioma cell lines for the susceptibility to wild type measles virus infection, which uses CD150 receptor for cell entry. We did not observe cytopathic effect (Fig. 4A) or viral particle production after infection of glioma cells with wt MV (Fig. 4B), in contrast to the laboratory MV strain, which uses the ubiquitously expressed CD46 as entry receptor (Fig. 4C). These results confirmed that in glioma cells CD150 is expressed in the cytoplasm, but not on the cell surface. The lack of CD150 cell surface expression could be linked to (i) the disturbed CD150 vertical segregation from cytoplasm to the cell surface, (ii) aberrant transcription of mCD150, or (iii) the presence of an alternative CD150 isoform(s).

### In glioma cells CD150 is colocalized with the endoplasmic reticulum and Golgi complex

**Fig 5:**
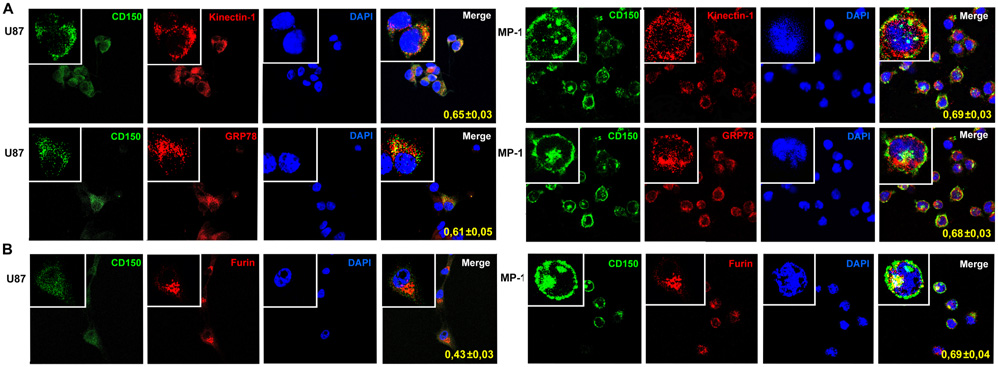
Colocalization of CD150 with the markers of endoplasmic reticulum and Golgi apparatus. U87 or MP-1 cells were stained for CD150 (green) and the markers of endoplasmic reticulum (A) or Golgi apparatus (B) (red). Markers for endoplasmic reticulum—kinectin-1 and GRP78, for Golgi—furin. Nuclei were visualized by staining with DAPI (4’,6-diamidino-2-phenylindole). Colocalization coefficients were determined using the Manders algorithm (which ranges from 0 to 1, where 0 is defined as no colocalization and 1 as perfect colocalization) and are indicated within the panels. Confocal microscopy shows the similar high colocalisation of CD150 and ER markers in both types of cells, but significantly lower colocalisation of CD150 and Golgi marker in glioma cell line U87 comparing to B cell line MP-1. Microscopic magnification of 630× was used for all pictures. Digital magnification of 3150× was made for the insertions. The data are presented as mean ± SD (n = 7).

To follow the intracellular trafficking of CD150 receptor, we analyzed the colocalization of CD150 with the markers of endoplasmic reticulum (ER) and Golgi apparatus. We studied CD150 intracellular distribution in glioma cell line (U87) in comparison with B lymphoblastoid cell line (MP-1) where it is expressed on the cell surface. For this, U87 and MP-1 cells were stained simultaneously with antibodies to CD150 and markers of endoplasmic reticulum (kinectin-1, GRP78) or Golgi apparatus (furin), followed by confocal microscope analysis (Fig. 5). Colocalization analysis was performed using the Manders coefficient, which revealed that in glioma cells, similarly to B cells, CD150 was colocalized with the endoplasmic reticulum for both ER markers: kinectin-1 (Manders coefficients 0.65±0.03 and 0.69±0.03 respectively; n = 7) and GRP78 (Manders coefficients 0.61±0.05 and 0.68±0.03 respectively; n = 7). However, CD150 was colocalized with the Golgi marker furin in the glioma cell line with a colocalisation coefficient significantly lower than that for the MP-1 B cell line (Manders coefficients 0.43±0.03 and 0.69±0.04 respectively; n = 7; p = 0.00003), suggesting that the entry of CD150 into Golgi complex is different in the two analysed cell types. Therefore, in glioma cell line the transport of CD150 protein from ER to Golgi could be altered that may partially explain the cytoplasmic localization of CD150 in glioma cells.

### Expression of CD150 domains at the mRNA level in glioma cell lines

To check the domain structure of CD150 from glioma cell lines, RT-PCR was performed with several sets of primers, designed to detect the exons encoding the different parts of CD150 receptor (extracellular, transmembrane and cytoplasmic).

**Fig 6:**
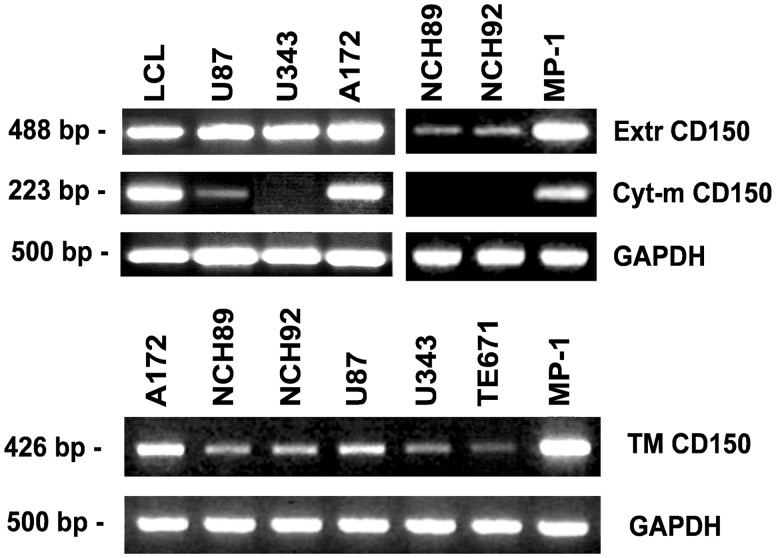
Expression of different domains of CD150 in glioma cell lines. Specific primers to the extracellular (Extr CD150), cytoplasmic (Cyt-m CD150) and transmembrane plus cytoplasmic (TM CD150) parts of CD150 were used for RT-PCR analysis of CD150 expression. LCL and MP-1 cells were taken for positive control. The quality and quantity of cDNA was monitored by GAPDH expression. CD150 extracellular and transmembrane domains on mRNA level were detected in all studied glioma cell lines, while cytoplasmic domain was found only in U87 and A172 cell lines. One of more than five representative experiments.

Exons encoding the extracellular part of CD150 (Extr CD150) were detected in glial cell lines U87, U343, A172, TE671 and in primary glioma cells NCH89 and NCH92 (Fig. 6). Exons encoding CD150 conventional cytoplasmic domain that is present in mCD150, as well as in cCD150 and sCD150 isoforms (Cyt-m CD150), were expressed only in U87 and A172 cells. PCR with the sense primer in the exon encoding transmembrane domain and the antisense primer downstream of the non-coding RNA region, which recognize mRNA of splice isoforms with transmembrane region and any cytoplasmic tail variants, showed that all studied glial cell lines expressed CD150 transmembrane domain (TM) (Fig. 6). Thus, all tested glioma cell lines have CD150 extracellular and transmembrane domains that should allow its cell surface expression unless it lacks the leader sequence.

### Identification of a novel CD150 splice isoform

**Fig 7:**
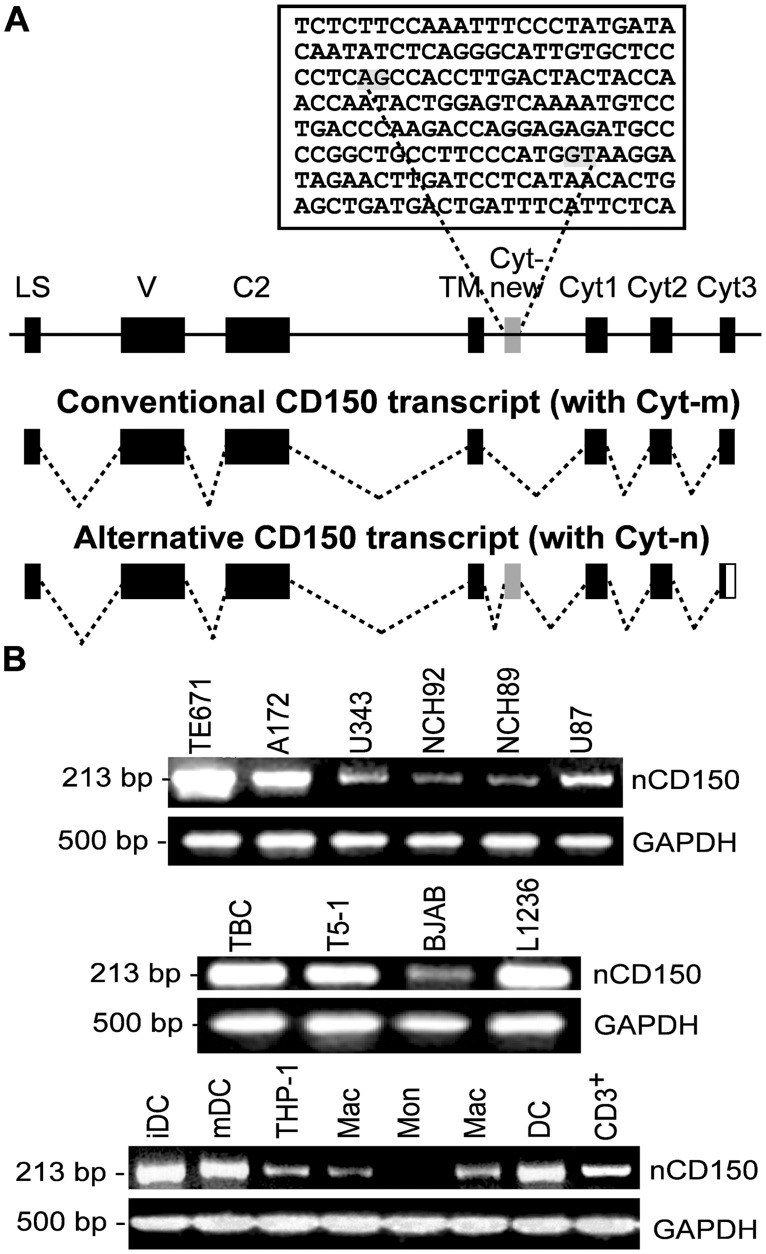
The expression of a novel CD150 splice isoform (nCD150). (A) Schematic representation of the CD150/SLAM gene structure and alternative splicing of mRNA. The exon designated Cyt-new is flanked with canonical splice sites (AG/GT marked in grey in the above genomic sequence). Exons are shown by filled rectangles, noncoding sequences—by solid line. Abbreviations: LS—leader sequence, V and C2—extracellular domains, TM—transmembrane domain, Cyt—cytoplasmic tail. (B) Expression of a nCD150 splice isoform was found in TE671, A172, U343, NCH92, NCH89 and U87 glioma cell lines, human tonsillar B cells (TBC) and cell lines of B cell origin, human acute monocytic leukemia cell line THP-1 and dendritic cells (DC), T cells (CD3^+^), monocytes (Mon) and macrophages (Mac), but it was not detected in human primary monocytes (Mon). The quality and quantity of cDNA was monitored by GAPDH expression. One of three representative experiments.

In several RT-PCR sets with TM primers we were able to get a PCR product of approximately 500 bp length from U87 mRNA. The expected size of the PCR fragment with TM primers for mCD150 splice isoform is 426 bp, as we previously showed for LCLs MP-1 B lymphoblastoid cell line [11]. The sequence of RT-PCR product that was obtained using U87 mRNA revealed the CD150 fragment with an insert of 83 bp between the TM and Cyt1 exons (Fig. 7A).

To determine if *SLAMF1* is aberrantly transcribed in the U87 cell line, cDNA from U87 cells was amplified using specific primers to the whole coding sequence of *SLAMF1*, cloned into pCI-neo vector, and sequenced. Two variants of full-length cDNA were found among the obtained clones. The first was identical to the conventional mCD150 cDNA. The second was a novel CD150 transcript with an 83 bp insert between TM and Cyt1 exons (Fig. 7A). The sequence of this transcript was submitted to GenBank under accession number BankIt1650007 CD150v3 KF471075. Both variants of cDNA did not contain any mutations, and the leader sequence was present and not modified.

Alignment with the human genomic sequences demonstrated that the novel transcript contains a previously unrecognized exon located 510 bp downstream of the exon for the transmembrane region. The exon designated Cyt-new is flanked with canonical splice sites AG and GT (Fig. 7A). The insertion of the Cyt-new exon results in the reading frame shift that leads to the formation of a premature stop codon in the Cyt3 and production of a novel isoform (nCD150) with cytoplasmic tail lacking any known signaling motifs. The predicted cytoplasmic tail of the nCD150 counts 94aa, while the mCD150 cytoplasmic tail is 72aa in length.

To determine whether this exon is specific for human *SLAMF1* genes, we searched for the homologs in the EST (expressed sequence tag) and genomic databases. The search demonstrated the presence of Cyt-new exon in the *SLAMF1* of primate species, including marmoset and tarsier, but not in the genomes of other mammals, demonstrating that the novel exon is a specific feature of primate *SLAMF1* gene. The sequence comparison showed however that, if there is any functional role for nCD150 isoform, it may be restricted to humans, great apes and gibbons. In these species the nucleotide sequence of the novel exon is 98% identical. In other examined primate species the exon contains either stop codon or frameshifts (S1 Fig).

### nCD150 expression

Specific primers for nCD150 were designed to follow up nCD150 mRNA expression in human cells of different origin, including primary hematopoietic cells and cell lines. We found nCD150 mRNA in all studied glial cell lines and in the primary glioma cells NCH89 and NCH92 (Fig. 7B). nCD150 mRNA was also detected in primary human tonsillar CD38^+^ B cells, lymphoblastoid cell line T5–1, Burkitt’s lymphoma cell line BJAB, and Hodgkin’s lymphoma cell line L1236 (Fig. 7B). Cells of human acute monocytic leukemia cell line THP-1, normal T cells (CD3^+^), primary dendritic cells and macrophages from peripheral blood, but not monocytes, expressed nCD150 mRNA as well (Fig. 7B). The specificity of all bands presented on Fig. 7B was verified by sequencing.

**Fig 8:**
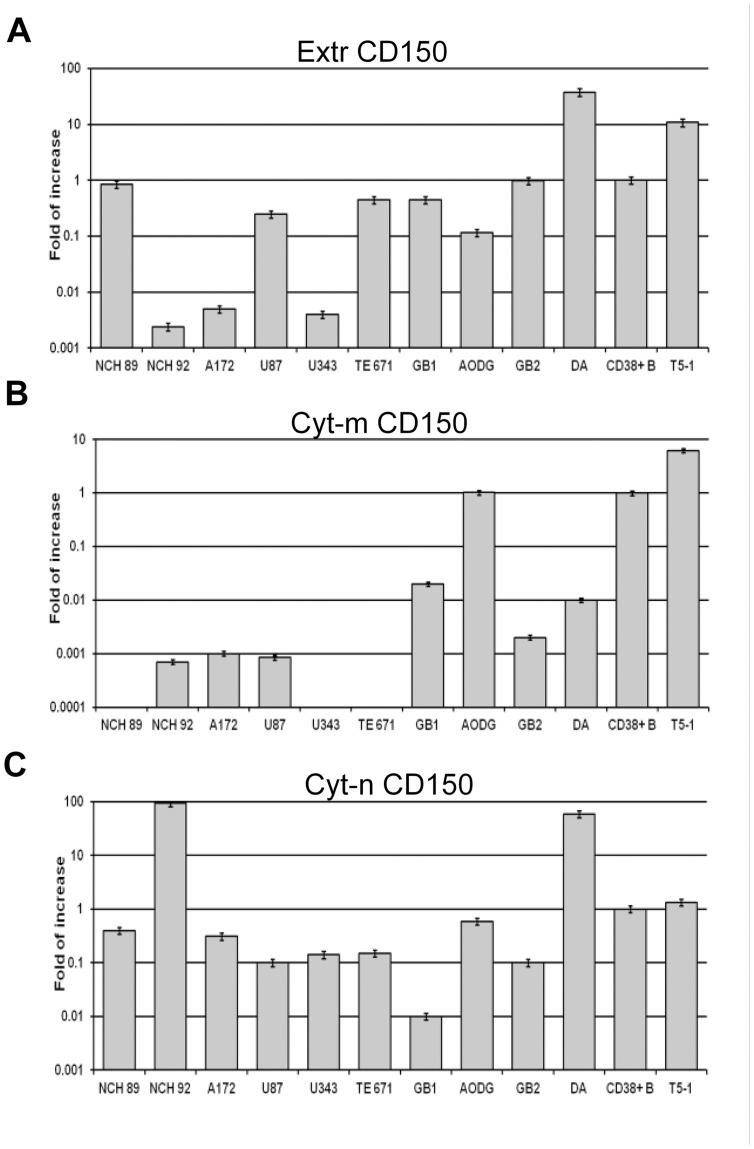
Real-time-PCR analysis of CD150 splice isoforms expression in glioma cell lines and primary tumors. We used 6 glioblastoma cell lines, four glioma tumor samples (GB1, GB2—glioblastoma, AODG—anaplastic oligodendroglioma, DA—diffuse astrocytoma), human tonsillar CD38^+^ B cells and lymphoblastoid cell line T5–1 for the analysis. Expression level of mRNA coding for each CD150 isoform was calculated using (ddCt) method, normalized to TBP and then expressed relative to respective isoform in CD38^+^ B cells, the value for which was set at 1. The results, presented as mean of triplicates (±SEM), are from one of three independent experiments. In glioma cell lines and glioma primary tumors the novel CD150 isoform is expressed at the high level, while the isoforms with the conventional cytoplasmic tail are absent or detected at the low level.

To access the quantitative ratio of mRNA expression level of different CD150 splice isoforms with conventional (Cyt-m—mCD150, sCD150, cCD150) or alternatively spliced (Cyt-n—nCD150) cytoplasmic tails we performed real-time PCR. It was shown that Cyt-n mRNA was expressed in all tested samples and at high level in primary glioblastoma cells NCH92 and diffuse astrocytoma tumor sample DA (Fig. 8C). In contrast, expression of CD150 splice isoforms’ mRNAs encoding conventional cytoplasmic tail (Cyt-m) was very low or practically undetectable with the exception of AODG primary tumor sample (Fig. 8B). Thus, we found that the expression pattern of CD150 splice isoforms mRNA in glioblastoma cell lines and tumor samples was different from primary B cells and LCL T5–1, as it was characterized by high level of nCD150 expression and no/low level of mRNA encoding CD150 splice isoforms with conventional cytoplasmic tail. Interestingly, seven out of ten tested glioma cell samples express the extracellular part of CD150 (Extr CD150) at the level comparable with B cells (Fig. 8A). Taken together, these data indicate that in glioma cells nCD150 is the predominant CD150 isoform.

**Fig 9:**
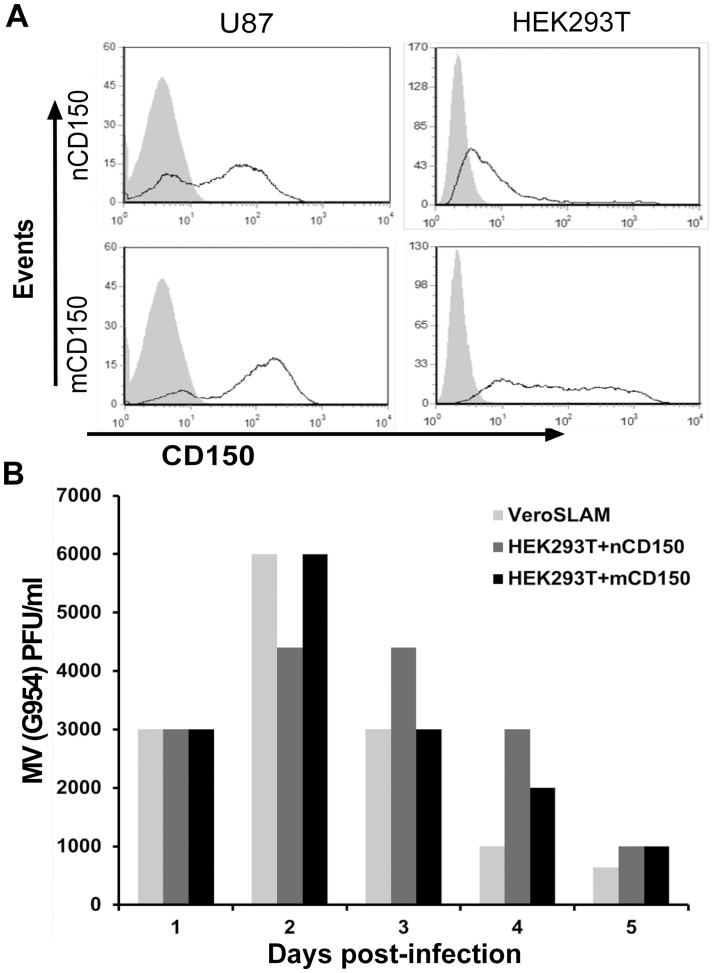
Cell lines transfected with mCD150 and nCD150 are susceptible to wild type measles virus infection. (A) Surface expression of mCD150 and nCD150 isoforms in HEK293T and U87 cell lines after transfection with respective plasmids measured by flow cytometry, after staining with anti-CD150 mAb IPO3. (B) Wild type MV (G954 strain) production in HEK293T cells transfected with mCD150 and nCD150, determined daily by plaque assay on Vero-SLAM cells, 24 h (1), 48 h (2), 72 h (3), 96h (4) and 120 h (5) post-infection. Vero-SLAM and non-transfected HEK293T cells were used as positive and negative control respectively. One of three independent experiments. The expression of both mCD150 and nCD150 isoforms on the surface of transfected cells allows the entry of wt measles virus to the cell.

Our results demonstrated that CD150 protein remains in the cytoplasm of glial cells (Figs. 3A, 3B, 4), although both splice isoforms, mCD150 and nCD150, contain a leader sequence and a transmembrane domain that are essential for the receptor’s surface expression. Overexpression upon transfection of U87 and HEK293T cell lines with either nCD150 or mCD150 isoforms resulted in cell surface expression of both CD150 isoforms in these cell lines (Fig. 9A). Moreover, similarly to mCD150, the expression of nCD150 on cell surface allowed wt MV entry to the cell (Fig. 9B).

## Discussion

Genome instability is underlying the key hallmarks of cancer. This often results in an aberrant gene expression profile in cells of different histogenesis. CNS tumors are characterized by numerous genetic and epigenetic abnormalities that allow classifying gliomas into distinct molecular subgroups. Some well-established molecular markers in CNS tumor subclassification are associated with chromosome 1 and include 1p/19q co-deletion, complete or partial 1p loss and frequent gain of 1q [28]. The mechanism of gene alteration due to chromosome 1 changes is largely unknown. Several genes on chromosome 1 have been implicated, but none have been clearly demonstrated to be the key players in glioma development. Among others, genes encoding a number of families of cell surface receptors are located on the long arm of chromosome 1, particularly the SLAM family. Here, for the first time, we report the expression of CD150/SLAMF1 in CNS tumors.

CD150 was first described as an antigen of activated lymphocytes [7,29,30], but the expression and functions of this antigen outside of the hematopoietic system were not explored. Previously, using immunohistochemical approaches, we found that CD150 was expressed in several tumors of ectodermal origin (e.g. squamous cell carcinoma of uterine cervix, rectum and oral cavity, basalioma), but not in their normal counterparts [31]. In the current study CD150 expression was shown in malignant cells of CNS tumors (Figs. 1A, 2). Since CD150 was not detected in normal brain tissues (Figs. 1B, 2) its expression could be considered as a potential diagnostic marker for CNS tumors.

Our immunohistochemical, flow cytometry, and immunofluorescent microscopy studies clearly showed CD150 expression in tumor cell cytoplasm, but not on the cell surface (Figs. 3A-B and 4). RT-PCR analysis detected CD150 transmembrane domain in all tested glioma cells (Fig. 6). This allowed us to propose that in glioma cells CD150 could be aberrantly transcribed, or a novel splice isoform with alternative cytoplasmic tail is present in these cells. Indeed, we identified a novel splice isoform—nCD150, with 83 bp insert between the transmembrane and cytoplasmic domains (Fig. 7A), resulted from alternative splicing, which integrated an additional previously unrecognized exon Cyt-new into the reading frame (Fig. 7A). It should be emphasized, that the nCD150 was expressed in all studied glioma cells, as well as in the majority of hematopoietic cells tested (Fig. 7B).

The absence or modification of the leader sequence in glioma cells could potentially lead to the cytoplasmic expression of CD150 splice isoforms in these cells. We obtained the full sequences of mCD150 and nCD150 from U87 cells. Both mCD150 and nCD150 splice isoforms did not contain mutations in the extracellular part and had the original leader sequence, and could thus be exposed on the cell surface. Indeed, transfection of mCD150 and nCD150 to HEK293T or U87 cells resulted in the surface expression of CD150 receptor (Fig. 9). Using densitometry analysis of CD150 western blots normalized to actin level, we estimated the relative levels of CD150 expression in different cell lines, including cell lines with CD150 overexpression. In different glioma cell lines CD150 expression levels were 7–26 times lower than in B-lymphoblastoid cell lines with CD150 cell surface expression. CD150 overexpression in glioma cell lines resulted in upregulation of CD150 expression from 20 to 110 times (in different subclones) above the endogenous level. Therefore, it is highly possible that CD150 relocalization to the cell surface depends on its expression levels. CD150 surface recruitment in glial cells may be affected by other intracellular mechanisms. Confocal microscopy studies of CD150 compartmentalization in glial cells demonstrated that CD150 localized both in endoplasmic reticulum and Golgi complex, however the colocalization coefficient for Golgi was lower than for ER (Fig. 5). Moreover, CD150 colocalization coefficient for Golgi in glial cells was significantly lower than in B cell line with high level of surface CD150 expression. This may reflect the lack of CD150 exposure on glioma cell surface. In dendritic cells CD150 is colocalized with Lamp-1 positive lysosomal compartment, and, upon ligation, is co-transported with acid sphingomyelinases to the cell surface. This is followed by formation of ceramide-enriched membrane microdomains, which promote vertical segregation of CD150 from intracellular storage compartments [32]. Glial tumors are characterized by low levels of ceramide that is associated with malignant progression, poor prognosis, and resistance to chemotherapy [33,34]. Thus, the cytoplasmic localisation of CD150 in glioma cells could also be explained by a disturbed CD150 vertical segregation due to an impediment in the ceramide metabolism.

On the other side, the level of CD150 glycosylation also contributes to its surface exposure in glioma cells, since inhibition of glycosylation downregulates the CD150 cell surface expression [7]. N-glycosylation starts in ER, and is followed by specific processing in Golgi, where oligosaccharides are replaced and/or modified in a strict order and manner. Surface receptors in Golgi acquire their specific oligosaccharide structure and composition, which would be recognized as a signal for their transport to the plasma membrane [35]. Tumor cells, including gliomas, have an altered pattern of protein glycosylation compared to normal cells due to the modifications of glycosylation machinery in the Golgi apparatus [36]. As mentioned above, CD150 colocalization with Golgi marker was significantly lower in glioma cells in comparison to B cell line (Fig. 5). Therefore, in glioma cells CD150 glycosylation could be insufficient or altered leading to the lack of its surface expression on the plasma membrane. This is supported by our results of western blot analysis that showed differences in the pattern of CD150 protein bands between glioma and B cells with preferential expression of bands with lower molecular weight in glioma cells (Fig. 3C). In MP-1 cell line the band of 40 kDa (Fig. 3C) corresponds to non-glycosylated protein core of mCD150 [7]. Moreover, multiple bands in glioma cells may correspond to different CD150 isoforms (sCD150, cCD150 or vmCD150/tCD150) [4, 10, 12], and/or previously not identified CD150 splice variants.

Our data also reveal another aspect of CD150 expression in glioma cells. Due to the lack of CD150 cell surface expression, glioma cell lines are resistant to wild type MV entry and consequent oncolysis (Fig. 4). Potential promotion of CD150 vertical segregation from intracellular storage compartments in glioma cells could open new perspectives for CD150-targeted, MV based oncolytic therapy of CNS tumors. Furthermore, utilization of retargeted MV, already used in clinical assays [23–26], allows virus entry into tumor cells, and may potentially lead to the interaction between MV hemagglutinin with intracellular CD150 and consecutive signaling. Indeed, recent report underlined the importance of CD150 signaling in the MV-induced tumor regression [37]. In this context, it is important to explore aspects of CD150 functions in glioma cells that is the subject of our current studies.

Despite the progress in discovery of novel glioma biomarkers, there is an urgent need for additional objective molecular markers that will help to refine the histomolecular classification of CNS tumors. Our studies revealed a novel CD150 isoform that is a specific feature of primate genomes and is a new potential molecular marker of CNS tumors. Broad studies of CD150 expression in CNS tumors in conjunction with evaluation of clinical outcomes will give an answer whether CD150 expression could be used as a reliable diagnostic, prognostic and predictive marker, as well as a target for the CNS tumors therapy.

## Supporting Information

S1 Fig Alignment of nucleotide sequences of the SLAMF1 Cyt-new exon of primate species: chimpanzee (Pan troglodytes), gorilla (Gorilla gorilla), orangutan (Pongo abelii), gibbon (Nomascus leucogenys), green monkey (Chlorocebus sabaeus), macaque (Macaca fascicularis), baboon (Papio Anubis) and marmoset (Callithrix jacchus). Deduced amino acid sequence of human nCD150 is shown above. Dots indicate nucleotide identity. Grey boxes show AG/GT splice signals. Hyphen indicates a gap introduced to maximize homology. Highlighted are stop codons.
(TIF)
