# Supplementary figures and images for: Expression of CD150 in Tumors of the Central Nervous System: Identification of a Novel Isoform
Source: PLoS One. 2015 Feb 24;10(2):e0118302. doi: 10.1371/journal.pone.0118302 (PMC4339833; doi:10.1371/journal.pone.0118302)

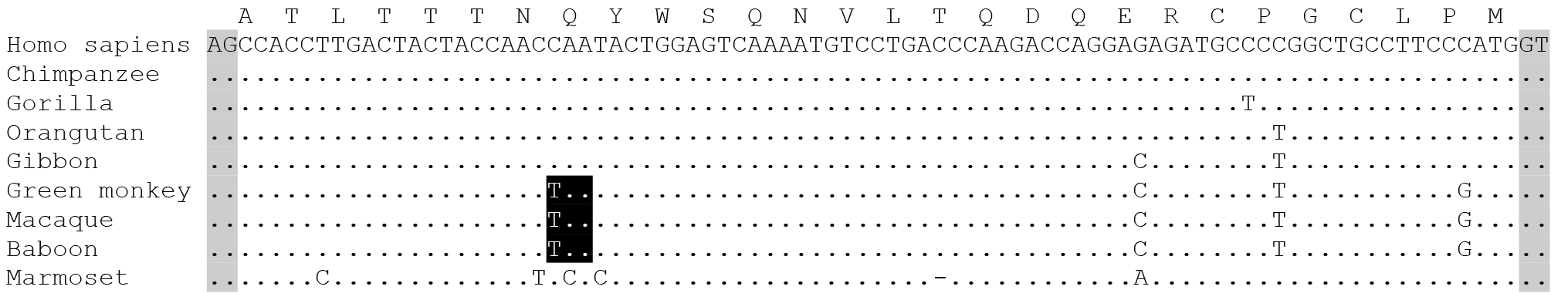

Supplement: S1 Fig — chimpanzee (Pan troglodytes), gorilla (Gorilla gorilla), orangutan (Pongo abelii), gibbon (Nomascus leucogenys), green monkey (Chlorocebus sabaeus), macaque (Macaca fascicularis), baboon (Papio Anubis) and marmoset (Callithrix jacchus). Deduced amino acid sequence of human nCD150 is shown above. Dots indicate nucleotide identity. Grey boxes show AG/GT splice signals. Hyphen indicates a gap introduced to maximize homology. Highlighted are stop codons. (TIF) [file pone.0118302.s001.tif]
